# Studying the Effect of Sertraline in Reducing Aggressive Behavior in Patients with Major Depression

**DOI:** 10.15171/apb.2017.033

**Published:** 2017-06-30

**Authors:** Alireza Farnam, Arezoo MehrAra, Hossein Dadashzadeh, Golamreza Chalabianlou, Salman Safikhanlou

**Affiliations:** Research Center of Psychiatry and Behavioral Sciences, Razi Hospital, Tabriz University of Medical Sciences, Tabriz, Iran.

**Keywords:** Depression, Aggression, Sertraline, STAXI-II

## Abstract

***Purpose:*** Depression causes dysfunction in various spheres of individual and social life, which is now considered as the fourth-leading cause of the global disease burden. Given that violence and aggression associated with depression in the community cause serious damage to the family, the prediction, early detection and effective treatment of aggressive and violent behavior are essential. The present study compared the severity of aggression before and after treatment with sertraline in patients with major depression.

***Methods:*** This is an intervention type study and the study population consisted of patients with depression and aggression. The sampling included 23 eligible patients. Data were obtained by SCID-I, SCID-II, STAXI-II, BDI-II and was also analyzed using SPSS 23 software.

***Results:*** The results showed that depression, anger mood, desire to verbally express anger, controlling anger and anger control before treatment was reduced but the desire for physical expression of anger increased.

***Conclusion:*** Obtained results in this research support the effect of Sertraline on reduction of severity of depression, reduction of severity of symptoms of aggression and anger (state of anger, anger feeling, and the tendency to express anger verbally), increased controlling external anger and significantly controlling internal anger. Hence, Sertraline can be found effective in the treatment of patients with depression and aggressive behaviors. Also Sertraline increases tend to cause physical representation of anger, then this issue supports the increase in the euthanasia behavior in primary days of treatment with SSRI that requires more assessments.

## Introduction

Mental disorder is an illness with psychological and behavioral protests which cause considerable confusion in the different functional areas and is developed as a result of biological, social, psychological, genetic, physical or chemical disorders. According to the revised version of the fourth edition of the Diagnostic and Statistical book of DSM-IV-TR, mental disorders contain 17 main categories (children's disorders, mood etc) and 375 disorders (major depression and schizophrenia etc.).^1^

It can be said that mental disorders are the greatest public health problems of the current era, such that depression has exceeded forecasts and has become the biggest cause of the disease.^2^

Depression occurs in about one of five women and one in ten men in a lifetime.^3^

In addition to the problems caused by depression, there is an idea that aggression plays a major role in depression development which has been fused in psychiatric thinking composition.^4^

Emotion and aggressive behavior is a common element of a whole hypersensitivity in response to any threat and tend to attack.^5^

Emotion and aggressive behaviors include a spectrum such that their common elements are hypersensitivity in response to any threat and tend to attack. In the mildest case, the spectrum starts with hypersensitivity and the increasing severity of the threats, verbal intimidation, physical violence and bullying is also followed by murder in the other side of the spectrum and sometimes lead to violence and sadism.^5^

Aggressive and violent behaviors are usually observed as symptoms of many psychiatric diseases. For example, violence has been seen in patients with substance abuse, personality disorders as well as mood and anxiety disorders.^6^

One of the most important adverse effects of violence and aggression in society is harm to families. Depending on what was noted, prediction, early detection and effective treatment of aggressive and violent behavior are essential. The first step in this direction is recognition of the nature, causes, symptoms and phenomena associated with aggression and violence.

In the conducted studies, the probability of domestic violence against women in Canada was 51% and the prevalence of purely physical violence against women in various cities is estimated between 2.7 to 18.6%.^7-9^

According to Fava and Rosenbaum (1998), anger, hostility and irritability are generally observed in patients with depressive disorder. Almost 1/5 (one fifth) of outpatient depressed patients are referred with symptoms of attack. Anger periods are usually associated with activation of the autonomic systems such as tachycardia, sweating, hot flash and compression of the chest. Depressed patients are considerably anxious and exhibit hostile rage with anger attacks than non-depressed and are more likely to have refrained criteria, dependent, narcissistic or antisocial and borderline personality disorder. In several studies, it was suggested that anger attacks significantly decrease with anti-depressant treatment. Anger attacks in 53 to 71% of outpatient depression treated with anti-depressants such as Fluoxetine, sertraline and imipramine disappeared. Moreover, the amount of anger attacks after treatment with Fluoxetine (7 to 6%) have no difference with the observed data after treatment with sertraline (8%) and imipramine (10%) and these values ​​are less than the incidence associated with placebo (20%). Finally, since the serotonergic neurotransmitter system is involved in the regulation of aggressive behavior in humans and animals, it can be assumed that anti-depressants affective on this system can be effective in depressed patients with dramatic aggression.^10^ In their previous study, Mezza et al. revealed the reduction of HR and HRV which confirms vagal activity in aggressive patients.^11^ In one study, it was found that there is a relationship between decrease in DBP, SBP and HR and increase in the aggressive behavior of preschool children.^12^ It was also found that SCL was higher in patients with a greater score of aggression, also anger triggers driving under the influence at the time of placement.^12^

In another study in 2013, Baker et al. concluded that reduction of skin conductance activity (SCA) in babies predict aggressive behavior at 3 years of age.^13^ Dibajnia and Moghadasin reported that numerous studies show the association between experience and expression of anger, depression, anxiety and stress.^14^ Researchers also reported that based on Pearson correlation coefficient and stepwise regression analysis of previous results the state of anger, trait of anger and internalized anger expression are the best predictors of depression. The scale of internal rage mood and anger expression respectively is the best predictor for anxiety and Trait Anger Scale, inner expression of anger and rage mode are also the best predictors of stress.^14^

Few studies have been conducted on aggression but according to their results, in an acceptable rate it can be concluded that low excitation of the autonomic nervous system is a biomarker of aggression.

## Materials and Methods

This study is an interventional type with a population of depressed patients having aggressive behaviors, referred to psychiatric clinics affiliated with the University of Medical Sciences during 2015 to 2016. The method used was simple random sampling and it included 23 samples.

### Inclusion criteria

This includes the following:

1) Diagnosis of depression with any degree and severity was based on the clinical evaluation of specialists and structured interviews based on the DSM-IV-TR criteria as well as the Beck and SCID questionnaires.^15-17^

2) Patients in the first period of depression or who previously did not receive any anti-depressant drug treatment.

3) The presence of a history of aggressive behaviors or physical examination was based on Trait Anger Expression Inventory (Anger scale score of 8 to 32).

4) Patients tend to use higher quality drugs, albeit with a higher cost.

5) Drug therapy using a Selective Serotonin Reuptake Inhibitor (SSRI) Asentra film-coated tablets (ACTOVERCO Pharmaceutical factory, Karaj, Iran, under license of KAKA, Slovenia) which started with a dose of 50 mg daily which increased to a dose of 100 mg daily later.

6) Having Iranian nation.

7) The age range of 20 to 40 years (due to the continuing development of CNS up to about 19 years old).

8) Patient agreement to participate in the study by signing an informed consent.

### Exclusion criteria

1) The existence of any current psychiatric disease or a history of depression exception.

2) The use of any drugs affecting the CNS or PNS other than anti-depressants.

3) The existence of any of these diseases: amyloidosis, acute porphyria, cardiovascular diseases, respiratory, endocrine, nervous system, malnutrition, abuse of alcohol and drugs, multiple sclerosis, amyotrophic lateral sclerosis (ALS) and other diseases such as primary and secondary autonomic conflict.

4) Pre-menstruation period for women.

5) The history of these diseases were also considered in patients and in the case of strong suspicion of a genetic or environmental predisposition, the patient was not enrolled.

6) It is noteworthy that during studying each individual who had lost one of the conditions and criteria or has one of the criteria or for whatever reason is not willing to continue to participate in the study or becomes unavailable is excluded and their data were not used in the statistical analysis.

## Results

The average age of participants was 33.91 with a standard deviation of 14.8 and the minimum and maximum ages of 21 and 40 years respectively. Mean age was 34.63 years with a standard deviation of 8.06 and the average age of men was 30.50 years old with a standard deviation of 8.81.

Among the age ranges of women and men, the difference was not significant (F=0.30, T=0.87, Df=4.13, P=0.44). As well as between women and men in terms of education, the difference was not significant (Chi-Sq=3.69, Df=1, P=0.06).

Table 1 shows the severity of depression before and after the drug treatment such that the severity of symptoms was reduced.

**Table 1:** Paired t-test for assessing changes in depression symptoms after treatment as compared to before treatment.

**Variable**	**Stage**	**Average**	**SD**	**t**	**P-Value**
**Beck depression**	Before treatment	25.32	10.20	3.34	0.003
	After treatment	18.39	10.22		

Additionally, classification of the patients in terms of severity of depression before and after treatment using sertraline was demonstrated in Table 2.

**Table 2:** Severity of depression symptoms in treatment stages, according to Beck.

-	**Before treatment**		**After treatment**	
**Depression level**	Number	%	Number	%
**Normal**	0	0	6	26.1
**Slight**	8	34.8	9	39.1
**Mild**	6	26.1	4	17.4
**Severe**	9	39.1	4	17.4

According to Table 2, depression severity after starting sertraline decreased significantly such that from 23 patients, 6 patients reported themselves with no depression symptom and others were also adjusted from extreme level to mid-level and upper-middle class moderate to mild level. The Wilcoxon test at 0.02 levels was significantly achieved and represented the loss of these symptoms and severity of depression.

Results of Table 3 showed the state of anger, feelings of anger, a desire to verbally express anger, control anger and internal anger control before starting the drug and after that was significant so that the severity of symptoms are reduced and anger control is also increased.

**Table 3:** Paired t-test for the difference between variables Trait Anger before and after the treatment of depression.

**Variable**	**Stage**	**Average**	**SD**	**t**	**P- Value**
**Anger state**	Before treatment	34.65	10.83	3.32	0.003
	After treatment	26.04	10.10		
**Anger feeling**	Before treatment	14	3.45	4.18	000
	After treatment	10.61	3.33		
**Tend to represent verbal anger**	Before treatment	12.30	4.73	3.01	0.01
	After treatment	8.78	4.13		
**Anger trait**	Before treatment	28	5.62	0.96	0.35 NS
	After treatment	26.65	5.74		
**Aggressive behavior**	Before treatment	11.09	3.80	1.53	0.14 NS
	After treatment	10.22	3.23		
**Aggressive reaction**	Before treatment	16.91	3.30	0.52	0.61 NS
	After treatment	16.43	3.69		
**Presence of external anger**	Before treatment	19.65	5.06	1.55	0.14 NS
	After treatment	18	4.95		
**The presence of internal anger**	Before treatment	20.74	3.29	1.17	0.25 NS
	After treatment	19.61	3.60		
**Control on external anger**	Before treatment	17.30	4.19	-4.44	000
	After treatment	21.87	4.51		
**Control on internal anger**	Before treatment	16.26	4.66	-4.06	0.001
	After treatment	21.70	4.88		

Since the variable, tendency to represent anger physically in the Kolmogorov- Smirnov test was significant and had no normal distribution, the Wilcoxon test was used. Results of Table 4 revealed that the tendency to represent anger physically in order to increase scores before starting the drug and after that was significant. It can be interpreted that although Sertraline reduced verbal anger representation effectively, it also increased the willingness to represent anger physically.

On the other hand, there was a significant positive correlation between anger state, anger trait, aggressive behavior, aggressive feeling, tend to represent anger verbally, and aggressive reaction.

Results of the multivariable regression in Table 4 demonstrated that aggressive feeling with correlation coefficient of R=0.43 and determination coefficient of R^2^=0.18 predicts that about %14 of the changes is related to the severity of symptoms of depression.

**Table 4:** Wilcoxon test for studying before- and after- test variable tending to represent anger physically.

**Model**	**Entered variables**	**Multidimensional correlation coefficient**	**Determination coefficient of R** ^ 2 ^	**Adjusted R**	**Scale error**	**Beta standard**	**t**	**Significance level**
1	Aggressive feeling	0.43	0.18	0.14	9.48	0.43	2.16	0.04

## Discussion

Our research confirmed the study of Fava and Rosenbum (1998) who concluded that Sertraline was effective in the reduction of aggressive attacks and controlling the anger and increased it significantly.^10^

On the other hand, according to Dibajnia and Moghadasian^14^ and Khakbaz et al^15^ a significant relationship between depression and aggression was observed.

Similar to the research of Fan et al.,^16^ our study also emphasized that Sertraline led to a reduction of depression symptoms and also anger after 8 weeks of treatment.

Strik and et al. ^17^ in their study concluded that Fluoxetine was effective in reducing the symptoms of depression and anger in patients with severe depression and we also found the same results using Sertraline.

Dibajnia and Moghadasian^14^ in their study reported anger state, internal anger expression and anger trait as the best predictors of depression and our study also revealed that anger feeling could predict 14% of depression.

## Conclusion

Obtained results in this research support the effect of Sertraline on reduction of severity of depression, reduction of severity of symptoms of aggression and anger (state of anger, anger feeling, and the tendency to express anger verbally), increased control of external anger and significantly increased control of internal anger. Hence, Sertraline can be found effective in the treatment of patients with depression and aggressive behaviors.

According to analysis, Sertraline increases tend to cause physical representation of anger, then this issue supports the increase in the euthanasia behavior in primary days of treatment with SSRI that requires more assessments.

Limitations of the study include small sample size and short period of treatment.

## Acknowledgments

All participants and subjects who helped in the collection and codification of this article are acknowledged.

## Ethical Issues

Not applicable.

## Conflict of Interest

The authors declare no conflict of interests.
